# Elevated INR in a COVID-19 patient after concomitant administration of azvudine and anticoagulants

**DOI:** 10.3389/fphar.2023.1191608

**Published:** 2023-05-19

**Authors:** Xi Zhang, Fengwei Jiao, Guangrun Li, Xiaojia Yu, Yuqing Pei, Ying Zhang, Zihui Wang, Pengfei Li

**Affiliations:** ^1^ Department of Pharmacy, Beijing Chao-Yang Hospital, Capital Medical University, Beijing, China; ^2^ Department of Respiratory and Critical Care Medicine, Beijing Institute of Respiratory Medicine and Beijing Chao-Yang Hospital, Capital Medical University, Beijing, China; ^3^ Department of Chinese Communist Youth League Committee, Beijing Chao-Yang Hospital, Capital Medical University, Beijing, China; ^4^ Research Ward/Phase I Clinical Trial Unit, Beijing Chao-Yang Hospital, Capital Medical University, Beijing, China

**Keywords:** azvudine, warfarin, rivaroxaban, DDI, international normalized ratio (INR)

## Abstract

**Background:** Azvudine (FNC) is a promising treatment candidate for managing coronavirus disease 2019 (COVID-19). However, drug interactions with azvudine have been poorly studied, especially with no reported cases of azvudine with anticoagulants such as warfarin and rivaroxaban.

**Case summary:** The patient was diagnosed with lower limb venous thrombosis and took warfarin regularly. The international normalized ratio (INR) was stable (2.0–3.0). However, the INR increased to 7.52 after administering azvudine. The patient had no other factors justifying this change. This increase in INR occurred again with the administration of azvudine in combination with rivaroxaban, and the INR increased to 18.91. After azvudine administration was stopped, the INR did not increase when rivaroxaban was used alone.

**Conclusion:** Azvudine, warfarin, and rivaroxaban might have previously unidentified drug interactions that increased the INR. Therefore, the INR must be closely monitored when they are concomitantly administered in COVID-19 patients.

## 1 Introduction

Azvudine (FNC) is the first double-target nucleoside drug, which has demonstrated significant and broad-spectrum *in vitro* antiviral effects against targets such as HIV (Peng et al., 2014), HBV (Peng et al., 2014), HCV (Smith et al., 2009), and EV71 (Xu et al., 2020). It is also a promising treatment candidate for managing coronavirus disease 2019 (COVID-19) (Ren et al., 2020). FNC is completely absorbed by passive diffusion and active transport mechanisms. P-gp, MRP2, and BCRP could influence the absorption of FNC in the small intestine (Liu et al., 2017). FNC is not metabolized by cytochrome P-450 (CYP); therefore, drug–drug interactions (DDIs) are less likely.

Warfarin is a direct oral anticoagulant and also a narrow therapeutic index drug that requires frequent dose adjustments to minimize both thrombosis and bleeding-related adverse events (Gallagher et al., 2011). Drug interaction is one of the multiple factors that alter individual drug responses and thus contribute to international normalized ratio (INR) instability (Witt et al., 2016).

Rivaroxaban is an oral direct factor Xa inhibitor and has been developed as a favored alternative to coumarin derivatives (Kvasnicka et al., 2017). Approximately 2/3 of rivaroxaban is metabolized by hepatic cytochrome P450 (CYP) enzymes 3A4/5, 2J2, and CYP-independent enzymes, while the remaining 1/3 is eliminated unchanged via the kidney, involving transporters in active renal secretion such as P-glycoprotein (P-pg) and breast cancer resistance protein (BCRP) (Mueck et al., 2014). Many drugs, including aspirin (Perzborn et al., 2015), fluconazole (Mueck et al., 2013), and amiodarone (Cheong et al., 2017), had been reported to interact with rivaroxaban, increasing the risk of bleeding.

However, no reports are found indicating that azvudine interacts with warfarin and rivaroxaban. Here, we report a case of elevated INR in a COVID-19 patient, when azvudine and these two anticoagulants were concomitantly administered.

## 2 Case summary

**FIGURE 1:**
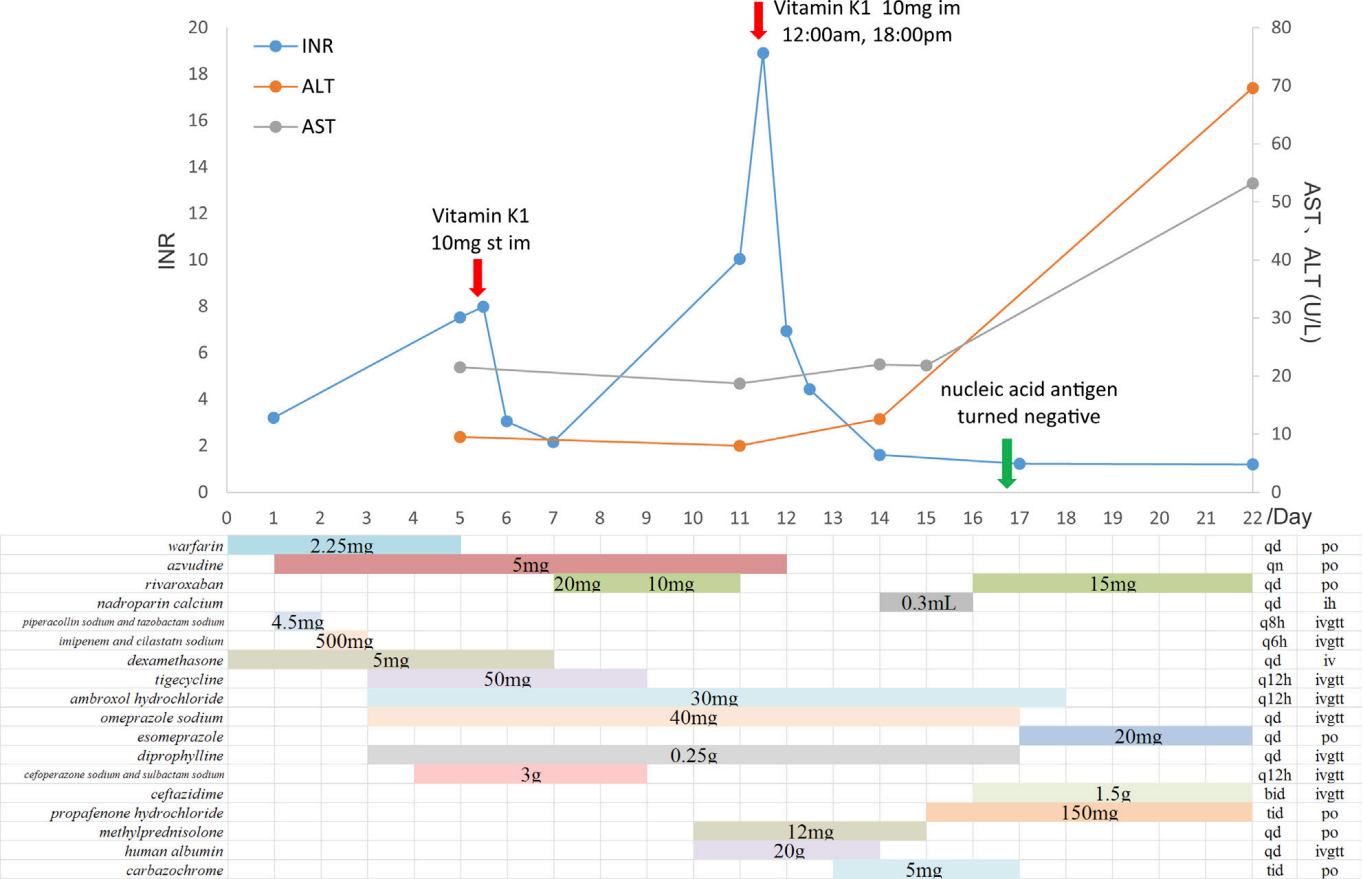
Time course of administration of azvudine and anticoagulants and changes in the INR and AST/ALT.

**FIGURE 2:**
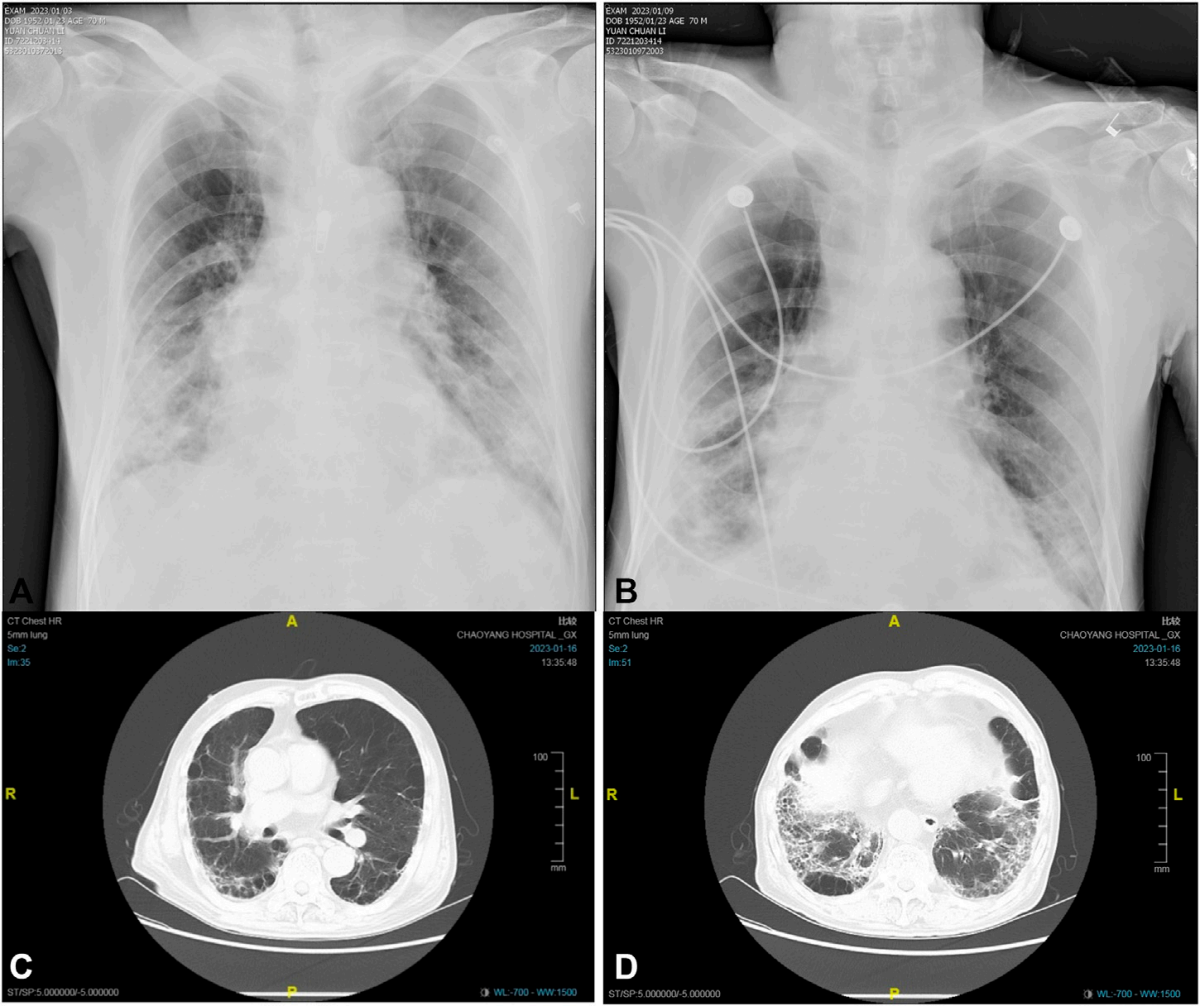
Dynamic chest X-ray and CT images of the patient:**(A)** the X-ray image on day 9, **(B)** the X-ray image on day 15, **(C)** the CT image on day 22, and **(D)** the CT image on day 22.

A 70-year-old man, diagnosed with fever, cough, and wheezing on December 23rd, was hospitalized in Fangshan Hospital of Traditional Chinese Medicine on December 26th (day 1). Laboratory results showed elevated C-reactive protein of 72.25 mg/L and WBC of 1.96 × 10^9^/L, and the chest CT image showed pneumonia and emphysema in both lungs. The patient was diagnosed with COVID-19 infection complicated with respiratory failure; he received azvudine (day 2) and dexamethasone; nasal catheter oxygen therapy (the oxygen flow rate was 3-4 L/min); and broad-spectrum antibiotics considering the accompanying bacterial infection (Figure 1). Given a past medical history of lower limb venous thrombosis, the patient took 2.25 mg of warfarin once a day, with the INR being monitored regularly (between 2.0 and 3.0). On day 4, the patient’s oxygenation index was as low as 78 mmHg, and he was transferred to Beijing Chaoyang Hospital for treatment. The baseline characteristics (day 4) are shown in Table 1. Relevant imaging materials are shown in Figure 2. The INR was 3.2, and the blood test showed that his liver function was normal on day 4. Due to deterioration in the condition, warfarin, azvudine, and dexamethasone were administered continuously, and broad-spectrum antibiotics were still used but adjusted to tigecycline, ambroxol hydrochloride, omeprazole, and diprophylline, which were added on day 4; in addition, high-flow nasal cannula oxygen therapy (FiO_2_ needed up to 90%) and intermittent prone positioning were applied. The detailed drug dosages and duration are shown in Figure 1. At 9 a.m. on day 5, the critical value showed that the INR was 7.52, and no hemorrhage was observed. The change in the INR is shown in Figure 1. Then, warfarin was discontinued, and vitamin K1 was given on day 5. The CRP was 21.6 mg/L and WBC was 4.4 × 10^9^/L on day 5; it was suggested that the infection was serious, and cefoperazone sodium and sulbactam sodium were added for anti-infection treatment later. However, the INR decreased to 2.16 on day 7. Then, rivaroxaban was added on day 8. On day 10, the WBC was 3.8 × 10^9^/L, and the oxygenation index of the patient increased to 156 mmHg, so antibiotics were stopped and the FiO_2_ was adjusted to 60%. At 4 a.m. on day 11, the critical value showed that the INR increased again to 10.04, rivaroxaban was discontinued, and VK1 was given immediately. However, the INR continued to increase, and by 9 p.m. on day 11, the INR returned to 18.91, and vitamin K1 was given again. Azvudine was discontinued the next day. At 6 a.m. on day 12, the INR decreased to 6.94 and continued to decrease until it reached 1.61 on day 14. The patient was given nadroparin calcium and rivaroxaban successively. Since then, the patient was managed with nadroparin calcium and rivaroxaban successively for anticoagulation therapy, and the INR did not increase again and remained at about 1.2. On day 16, laboratory tests showed that CRP was 0.9 mg/L, and WBC was 4.9 × 10^9^/L; the oxygenation index of the patient increased to 252 mmHg, the patient’s condition improved, the FiO_2_ was adjusted to 37%, and ceftazidime antiinfective therapy was administered. On day 17, the patient’s nucleic acid antigen turned negative. The patient’s condition improved significantly, and he was discharged on day 24.

**TABLE 1:** Baseline patient characteristics.

Subject		Normal range
Gender	Male	—
Age (years)	70	—
Body weight (kg)	65	—
Body temperature (°C)	36.0	—
Pulse (times per minute)	90	—
Respiratory frequency (times per minute)	25	—
Blood pressure(mmHg)	127/87	—
APACHE score	10	—
Serum creatinine (umol/L)	81.3	(53.0–115.0)
Serum albumin (g/L)	28.6	(35.0–53.0)
WBC (×10^9^/L)	4.7	(4.0–10.0)
CRP (mg/L)	21.6	(0.0–10.0)
Platelet (*10^9^/L)	173.0	(100.0–300.0)
Total bilirubin (umol/L)	6.5	(3.4–20.5)
AST (U/L)	21.5	(4.0–40.0)
ALT (U/L)	9.5	(4.0–40.0)
LDH (U/L)	310.6	(80.0–250.0)
INR	3.2	(2.0–3.0)
APTT (seconds)	44.7	(28.0–42.0)
D-dimer (mg/L)	0.39	(0.00–0.30)

Abbreviations: ALT, alanine aminotransferase; APTT, activated partial thromboplastin time; AST, aspartate aminotransferase; CRP, C- reactive protein; LDH, lactic dehydrogenase; WBC, white blood cell.

## 3 Discussion

### 3.1 Evaluation of the DDI between azvudine and warfarin

Factors that affect the INR include medication adherence, diet, disease (such as liver dysfunction), and concomitant use of drugs (Reddy et al., 2015). In this case, the patient was taking warfarin properly for 2 years, and his INR was stable at 2.0–3.0, demonstrating that his medication adherence was good. The increased INR was not due to the improving adherence to hospitalization. The patient did not change his diet and adhered to a warfarin-only diet while taking warfarin and azvudine. His liver function was also normal before and during admission. A retrospective study (Zou et al., 2020) evaluated the correlation between coagulation function and COVID-19 and reported that coagulation dysfunction is common in patients with COVID-19, especially fibrinogen and D-dimer elevation, and the degree of elevation is related to the severity of the disease. As the disease recovers, fibrinogen and activated partial thromboplastin time also returned to normal. However, the impact of COVID-19 on the INR has been very mild; as COVID-19 became more severe, the INR only increased from 1.01 in mild cases to 1.04 in severe cases. The initial INR of this patient was 3.2, which is due to the novel coronavirus disease possibly. Therefore, the INR increased to 7.98, and the correlation between increased INR and COVID-19 progression was not considered. The patient was treated with multiple drugs, including azvudine, piperacillin sodium and tazobactam sodium, imipenem and cilastatin sodium, dexamethasone, tigecycline, omeprazole sodium, and diprophylline. Although warfarin was associated with piperacillin sodium and tazobactam sodium, imipenem and cilastatin sodium, the INR continued to increase 1 or 2 days after discontinuation of the two drugs, so the interaction between warfarin and the two drugs was not considered. INR increase has been reported when high-dose dexamethasone (>40 mg/day) and warfarin are concomitantly administered (Sellam et al., 2007), which may be due to the action of high-dose synthetic glucocorticoids on Niemann–Pick C1-like 1 protein (Ito et al., 2019). However, the dose of dexamethasone in this patient was 5.0 mg once a day, which was too low to cause any DDIs. Although tigecycline could reduce warfarin clearance, it did not significantly change the effect of warfarin on the INR. Omeprazole is a moderate inhibitor of CYP2C19, which reduces warfarin metabolism when combined with warfarin. On the other hand, omeprazole is a CYP1A2 gateway agent, and when combined with warfarin, it reduces warfarin exposure and INR; research studies reported that the coagulation time was not significantly changed (Unge et al., 1992). There was no report about the interaction between diprophylline and warfarin. At 9 a.m. on day 5, the critical value showed that the INR was 7.52, but cefoperazone sodium and sulbactam sodium were added at 1 p.m. on day 5, so there was no temporal–causal relationship between the drug and the increase in INR. The effect of these drugs on INR was excluded. The INR increased to 7.52 after 3 days of administering azvudine. There was a reasonable time relationship between drug combination and INR elevation. Then, warfarin was discontinued and vitamin K1 was given. As needed to treat COVID-19, the patient continued to take azvudine. On day 7, the INR decreased again to 2.16. There was a temporal–causal relationship between the improvement time of adverse events and the discontinuation time of the combination of the two drugs. To avoid the risk of bleeding, warfarin was not subsequently resumed, so it was unclear whether the INR increase will occur again after warfarin and azvudine were taken orally simultaneously.

The metabolism of warfarin primarily involves CYP2C9, CYP3A4, CYP1A2, and CYP2C8 (Sekimoto et al., 2022). However, azvudine is not metabolized by the P450 enzyme and is not an inhibitor or inducer of the P450 enzyme. The interaction between the two drugs is unlikely to be due to drug metabolism. In terms of pharmacodynamics, warfarin can inhibit the biosynthesis of functional vitamin K-dependent (VKD) coagulation factors (Chen et al., 2020). Although no azvudine-induced bleeding or related adverse events has been reported to date. We suspect that maybe azvudine could affect the biosynthesis of active VKD coagulation factors with possible synergy with warfarin. However, there is no evidence, and further verification studies are needed at a later stage.

The Drug Interaction Probability Scale (DIPS) has been widely used as a criterion for determining adverse events caused by DDIs (Horn et al., 2007). By using the DIPS evaluation of the interaction between azvudine and warfarin, the result was “probable,” which supported an interaction possibility.

### 3.2 Evaluation of the DDI between azvudine and rivaroxaban

The patient started oral rivaroxaban on day 8. At 4:00 a.m. on day 11, the INR was 10.04, which increased again. Moore et al. (2015) reported that the INR is abnormally elevated several hours after an oral administration of rivaroxaban and decreased significantly after a few hours because of the drug properties of rivaroxaban. This situation is completely different from the principle of abnormal increase in the INR caused by an oral overdose of warfarin. At 9:00 p.m. on day 11 (17 h later), the INR did not decrease but continued to increase to 18.91. Therefore, it was excluded that the increase in INR was caused by rivaroxaban alone. The patient had good medication compliance during hospitalization and normal liver function. While taking rivaroxaban, the patient also took tigecycline, ambroxol hydrochloride, omeprazole, diprophylline, cefoperazone sodium and sulbactam sodium, and azvudine. Tigecycline is a *P-gp* substrate, and rivaroxaban increases the activity of *P-gp* (Liu et al., 2017); the combination did not lead to increased exposure to rivaroxaban. Ambroxol hydrochloride, omeprazole, and diprophylline were again combined with rivaroxaban on day 17, and no increase in the INR was observed. The INR increased 2 days after cefoperazone sodium and sulbactam sodium were discontinued. Concomitantly, azvudine had been taken in combination with rivaroxaban, and the INR increased; there was a temporal–causal relationship between the increase in the INR and the combination of the two medicines. The INR did not increase again on day 17 when rivaroxaban was taken alone. Rivaroxaban and azvudine were not used in combination again, so it was unclear whether the INR increase will occur again after rivaroxaban and azvudine were taken orally simultaneously.

As reported by previous articles about rivaroxaban (Fernandez et al., 2021), the types of interactions assessed were PK interactions mediated by CYP3A, *P-gp* modulators, and/or gastric pH modifiers, and PD interactions mediated by other antithrombotic agents and nonsteroidal anti-inflammatory drugs (NSAIDs) for interaction studies. Azvudine is not metabolized by the P450 enzyme and is a mild P-gp inducer (Liu et al., 2017), and when combined with rivaroxaban, there was no increase in rivaroxaban exposure, which further increased PT or INR. Azvudine is not a gastric pH modifier. Vitamin K does not affect the anticoagulant activity of rivaroxaban, so if the anticoagulant effect is reversed by rivaroxaban, procoagulant reversal agents such as prothrombin complex thickener, activated prothrombin complex thickener. or recombinant factor Vlla should be used. Vitamin K could not reverse clotting abnormalities caused by rivaroxaban overdose. In this case, rivaroxaban was discontinued and vitamin K1 was given, then INR decreased to 1.61 on day 14. We suspected that the interaction between azvudine and rivaroxaban affects the biosynthesis of active VKD coagulation factors, and this phenomenon is consistent with our speculation about the interaction mechanism between azvudine and warfarin. Chen et al. (2020) reported three ways to inhibit VKD carboxylation: inhibit vitamin K epoxide reductase (VKOR) (such as warfarin), inhibit vitamin K reductase (VKR) in vitamin K redox cycling (such as clofazimine), and inhibit vitamin K availability within the cells. Coagulopathy from drugs that inhibit the VKOR, but not vitamin K, can be rescued by administering vitamin K. So, it may be that azvudine inhibited VKOR and played a synergistic anticoagulant effect with rivaroxaban, but this evidence needs to be confirmed in further studies.

Finally, as described previously, using DIPS results, the interaction between azvudine and rivaroxaban was rated as “probable.”

## 4 Conclusion

Azvudine, warfarin, and rivaroxaban might have previously unidentified drug interactions that increased the INR. Therefore, the INR must be closely monitored when they are concomitantly administered in COVID-19 patients.

## Data Availability

The original contributions presented in the study are included in the article/Supplementary Material; further inquiries can be directed to the corresponding authors.
